# Development of KASP molecular markers and fingerprinting based on reduced representation genome sequencing of garlic

**DOI:** 10.3389/fpls.2026.1785664

**Published:** 2026-05-08

**Authors:** Qingqing Yang, Jide Fan, Xiaohui Song, Biwei Zhang, Yongqiang Zhao, Xinjuan Lu, Jie Ge, Canyu Liu, Mengqian Li, Guangyang Liu, Yi Feng, Feng Yang

**Affiliations:** Xuzhou Institute of Agricultural Sciences in Jiangsu Xuhuai District, Key Laboratory of Biology and Genetic Breeding of Sweetpotato, Ministry of Agriculture and Rural Affairs/National Agricultural Experimental Station for Soil Quality, Xuzhou, China

**Keywords:** fingerprinting, garlic, KASP, SNP, variety identification

## Abstract

In this study, a garlic DNA fingerprinting system based on single nucleotide polymorphisms (SNPs) was developed using representative cultivated garlic accessions. SNP markers were identified based on reduced-representation genome sequencing (GBS) data from 77 garlic accessions. A total of 26,701,817 raw SNPs were detected and subsequently filtered to obtain 7,006 high-quality candidate SNP loci. Among these, KASP primers were successfully designed for 4,297 loci, corresponding to an assay design success rate of 61.3%. Of these candidate loci, 30 generated reliable KASP genotyping results, from which 13 high-quality core SNP markers were finally selected through stepwise screening based on genotyping quality, marker polymorphism, and discriminatory ability. The average minor allele frequency (MAF), expected heterozygosity (He), and polymorphism information content (PIC) of these core markers were 0.235, 0.281, and 0.227, respectively. Based on these 13 core SNP markers, an SNP-based DNA fingerprinting system for garlic was established. Genotyping analysis of the 77 accessions showed that 73 could be effectively distinguished, whereas 4 accessions remained unresolved. Overall, this core SNP marker set showed effective discriminatory power for most garlic accessions and provides a practical basis for germplasm identification, variety protection, and molecular breeding in garlic.

## 1 Introduction

Garlic (*Allium sativum* L.) is an important specialty vegetable crop whose bulbs are rich in vitamins, amino acids, and mineral elements, offering considerable nutritional and medicinal value (Ge et al., 2025). Germplasm resources serve as the material foundation for crop breeding and a crucial guarantee for the sustainable development of agriculture in China. These resources encompass specific cultivars or materials, including released and local varieties, as well as wild accessions, important genetic stocks, and newly bred lines (Zhang et al., 2024). As a typical vegetatively propagated crop, garlic has undergone long-term cultivation, resulting in diverse germplasm types, abundant natural variation, and rich genetic backgrounds. Currently, over 600 garlic germplasm accessions are preserved in China, most of which are local varieties (Sun et al., 2020). Due to its asexual propagation and the inability to perform targeted hybrid breeding, garlic exhibits a wide range of natural variation and rich genetic diversity accumulated over prolonged cultivation. At present, garlic variety identification relies heavily on morphological characterization, which is time-consuming, environment-sensitive, and often lacks accuracy. This presents challenges in counterfeit seed detection, intellectual property protection, germplasm cataloging and preservation, as well as new variety certification and application. Therefore, precise identification and evaluation of garlic germplasm resources have become pressing needs.

Traditional identification methods based on morphological, ecological, and histochemical traits have considerable limitations (Mohanty et al., 2023). Genotyping-by-sequencing (GBS), as an emerging application of the second-generation DNA sequencing technology, is mainly used to identify single nucleotide polymorphisms (SNPs) that can serve for crop improvement (Martina et al., 2025; Vodiasova et al., 2025). This technology is widely used for analyzing population structure, genetic diversity, population history and adaptive evolution. For example, it is used to construct genetic maps and conduct phylogenetic analysis in crop and animal populations (Hu et al., 2025). In addition, GBS is not limited by the reference genome. A single sequencing run can generate a large number of SNP markers from the entire genome range. It has the advantages of simple operation, short cycle and low cost. It shows broad application potential in crops with large genomes and relatively insufficient genetic resources research (Kang et al., 2026).

Although a chromosome-level reference genome of garlic has become available since 2020, garlic possesses an extremely large and highly repetitive genome (~16.24 Gb), which makes whole-genome resequencing (WGS) of dozens of accessions costly and computationally intensive when sufficient depth is required for accurate variant discovery (Sun et al., 2020). Therefore, for the purpose of developing robust, cost-effective SNP markers applicable to routine germplasm identification and breeding, we adopted a reduced-representation sequencing strategy GBS, which has been widely used for rapid SNP discovery and genotyping in large-genome species with high sample throughput and reasonable cost. In garlic, previous studies also highlighted that GBS provides an effective compromise between marker density and cost due to the large genome size (Jia et al., 2023). We acknowledge that reduced-representation approaches preferentially sample genomic regions near restriction sites; however, we mitigated this bias by applying stringent filtering and by selecting loci with stable genotyping performance for downstream KASP assay development. This technology can quickly obtain a large number of genetic markers, and is widely used for analyzing population structure, genetic diversity, population history and adaptive evolution.

DNA fingerprinting, first developed by British scientists in 1986, offers advantages such as ease of detection, high marker polymorphism, and accuracy, making it a powerful tool for variety and line identification (Nybom et al., 2014). Single nucleotide polymorphisms (SNPs), referring to DNA sequence polymorphisms caused by single-base variations, are a third-generation molecular marker widely used due to their high throughput, rapid detection, and low cost (Li et al., 2023). A variety of high-throughput and cost-effective SNP genotyping platforms have been developed, including the GoldenGate assay (Fan et al., 2003), the Infinium platform (Steemers and Gunderson, 2007) and the KASPar system, which is based on competitive allele-specific PCR developed by KBiosciences (Livak et al., 1995). DNA fingerprints consist of specific DNA fragments or SNP loci with high individual resolution, analogous to human fingerprints (Trick et al., 2012; Tian et al., 2015). This technique has been successfully applied in various crops such as *Cymbidium ensifolium* L (Shen et al., 2025), cigar tobacco (Wang et al., 2021), rice (Yang et al., 2019), cucumber (Kahveci et al., 2021).

In this study, a set of SNP markers suitable for discriminating garlic (*Allium sativum* L.) germplasm resources was developed using the KASP genotyping technology. A total of 77 garlic accessions were selected for Illumina NovaSeq sequencing. Core SNP markers were identified by alignment with a reduced-representation genome, and their genetic relationships, genetic diversity, and population structure were subsequently analyzed. In addition, DNA fingerprint profiles were constructed based on the final core SNP markers, enabling discrimination of 73 out of the 77 garlic accessions, providing a scientific basis and valuable data support for genetic diversity analysis, cultivar identification, and molecular breeding of garlic.

## 2 Materials and methods

### 2.1 Plant materials and DNA extraction

A total of 77 garlic accessions, including commercial cultivars and local varieties, were used in this study. All materials were maintained at the Xuzhou Academy of Agricultural Sciences. Young leaves were collected from five healthy plants per accession, frozen in liquid nitrogen, and stored at −80 °C prior to DNA extraction.

Genomic DNA was extracted using a modified CTAB method. DNA quality and concentration were evaluated by agarose gel electrophoresis and NanoDrop spectrophotometry. Only samples with OD260/280 ratios between 1.8 and 2.0 and showing intact high-molecular-weight DNA bands were used for library construction. Detailed information on the garlic germplasm accessions is provided in **Supplementary Table 1**. All raw sequencing data have been deposited in the NCBI Sequence Read Archive (SRA) under BioProject accession number PRJNA1313812.

A total of 77 garlic accessions were initially included in this study. Due to the fact that 4 accessions could not be distinguished by the final reduced core marker set, the number of distinguishable accessions under this marker set was 73.

### 2.2 Library construction and reduced-representation genome sequencing

Reduced-representation genome libraries were constructed following the genotyping-by-sequencing (GBS) protocol. Briefly, 1 μg of high-quality genomic DNA from each sample was digested with the restriction enzymes EcoRI and NlaIII at 37 °C. The digested fragments were subjected to end repair, phosphorylation, and A-tailing, followed by ligation with Illumina sequencing adapters containing unique barcode sequences.

Adapter-ligated DNA fragments were purified using VAHTS DNA Clean Beads and enriched by PCR amplification. The amplified libraries were purified again using magnetic beads and assessed for fragment size distribution and concentration using an Agilent 2100 Bioanalyzer. Libraries showing a single, narrow peak were considered qualified for sequencing. Qualified libraries were sequenced on the Illumina NovaSeq 6000 platform using paired-end sequencing. Raw sequencing data were generated in FASTQ format for subsequent bioinformatic analysis.

### 2.3 SNP filtering and selection for KASP marker development

Raw SNP data obtained from GBS sequencing were filtered to identify high-quality loci suitable for KASP marker development. SNPs were retained according to the criteria (Li et al., 2023). First, SNPs with conserved flanking sequences and without additional polymorphic sites within 50 bp upstream and downstream of the target site were retained to ensure primer design feasibility. The retained SNPs were further filtered according to sequencing depth, genotype quality, missing rate, minor allele frequency, and biallelic status to obtain high-confidence loci. To ensure locus specificity, the flanking sequences of candidate SNPs were aligned to the garlic reference genome using BLAST, and only uniquely mapped loci were retained. Candidate SNPs were then further evaluated based on chromosomal distribution and KASP primer design feasibility. PIC values used for candidate SNP prioritization were calculated based on allele frequencies derived from GBS genotyping data. The retained loci were considered candidate SNPs for downstream KASP assay development and validation.

### 2.4 KASP amplification, genotyping and final marker selection

KASP amplification and genotyping were performed for candidate SNP loci selected for experimental validation. In this study, 30 markers generated reliable KASP genotyping data. KASP genotyping was performed using a total reaction volume of 10 μL, consisting of 2.5 μL of genomic DNA, 5 μL of 2× KASP Master Mix, and 2.5 μL of primer mix. PCR amplification was conducted using the following thermal cycling program: an initial denaturation at 95 °C for 10 min; 10 touchdown cycles of 95 °C for 20 s and 61-55 °C for 60 s (with a decrease of 0.6 °C per cycle); followed by 35 cycles of 95 °C for 20 s and 55 °C for 60 s.

For samples showing suboptimal genotyping performance, three additional amplification cycles of 95 °C for 20 s and 57 °C for 60 s were performed. After PCR amplification, fluorescence signals were collected and analyzed, and genotypes were assigned based on allele-specific fluorescence clustering. Based on the KASP genotyping results, marker statistics including missing rate, heterozygosity, and polymorphism information content (PIC) were recalculated. The 30 successfully genotyped KASP markers were further evaluated according to amplification stability, cluster clarity, and discriminatory power, from which 13 core markers were identified as the minimal marker set for DNA fingerprint construction and accession discrimination. Primers used for KASP validation and final marker selection are provided in **Supplementary Table 2**.

For accession discrimination, each garlic accession was represented by its multilocus genotype across the final core KASP markers, and multilocus genotype comparison was conducted following a standard genotype-based identification strategy as implemented in GenAlEx (Peakall and Smouse, 2012). Two accessions were considered indistinguishable when they showed identical genotypes across all informative loci in the final marker set. Loci with NN or missing genotype calls were not treated as discriminating sites.

### 2.5 Core SNP marker selection and population analysis

Allele frequencies for each KASP marker were calculated based on genotyping results. Polymorphism information content (PIC) values were computed using PIC Calc v0.6. SNP loci with relatively high PIC values, low missing rates, and stable genotyping performance were selected as core markers for DNA fingerprint construction (Hiremath et al., 2012).

Genotyping data from the selected core SNP markers were converted into binary format and combined to generate SNP-based DNA fingerprint profiles for the garlic accessions. Each accession was represented by a unique combination of SNP genotypes, enabling reliable discrimination among varieties.

In addition, the developed KASP-SNP markers were used for population genetic analysis. SNP genotyping data were imported into STRUCTURE v2.3.1 for Bayesian clustering analysis, with the number of assumed populations (K) ranging from 1 to 20. For each K value, 10 independent runs were performed using a burn-in period of 100,000 iterations followed by 500,000 Markov Chain Monte Carlo (MCMC) iterations. The optimal K value was determined using the ΔK method implemented in Structure Harvester.

Phylogenetic relationships among garlic accessions were inferred using the maximum likelihood method implemented in FastTree, and the resulting tree was visualized and edited using MEGA 7.0. Principal component analysis (PCA) was performed using Cluster 3 in combination with GenAlEx to further evaluate genetic relationships among accessions. Because the 13 core KASP markers were selected primarily for fingerprint construction, additional population genetic analyses were also performed using the 7,006 high-quality GBS-derived SNPs to obtain a more robust estimate of genetic structure and differentiation.

### 2.6 DNA fingerprint construction

Based on the SNP genotyping data, a Perl script was used to screen and determine the optimal SNP marker combination for variety fingerprint identification. The genotype information corresponding to the selected marker combination was displayed in a heatmap format, thereby constructing a DNA fingerprint map. The horizontal axis represents different samples, and the vertical axis represents each SNP locus. To facilitate intuitive distinction, different genotypes were labeled with differentiated colors: C/C - Yellow, A/A - Green, T/T - Blue, G/G - Purple, A/T - Orange, A/G - Pink, A/C - Dark Pink, T/G - Brown, T/C - Dark Green, C/G - Light Blue, C/T - Gray, G/T - Deep Red, N/N - White (genotype absence).

## 3 Results and analysis

### 3.1 Sequencing data quality assessment

A total of 918.00 Gb of clean data were generated from the 77 garlic accessions using the Illumina NovaSeq platform. The average clean data yield per sample was 11.92 Gb, indicating sufficient sequencing throughput for downstream SNP discovery. The GC content of the clean reads ranged from 35.98% to 38.72%, with an average of 37.69%, showing a relatively narrow distribution without obvious GC bias (**Supplementary Table 3**). The average sequencing depth across target loci was 8.15× per sample, ranging from 3.82× to 16.06×. The overall mapping rate to the reference genome ranged from 90.91% to 99.79%, with an average of 97.77%, suggesting that most samples exhibited high alignment efficiency and reliable genomic representation for SNP detection.

### 3.2 Polymorphic SNP Loci analysis in garlic accessions

**Figure 1:**
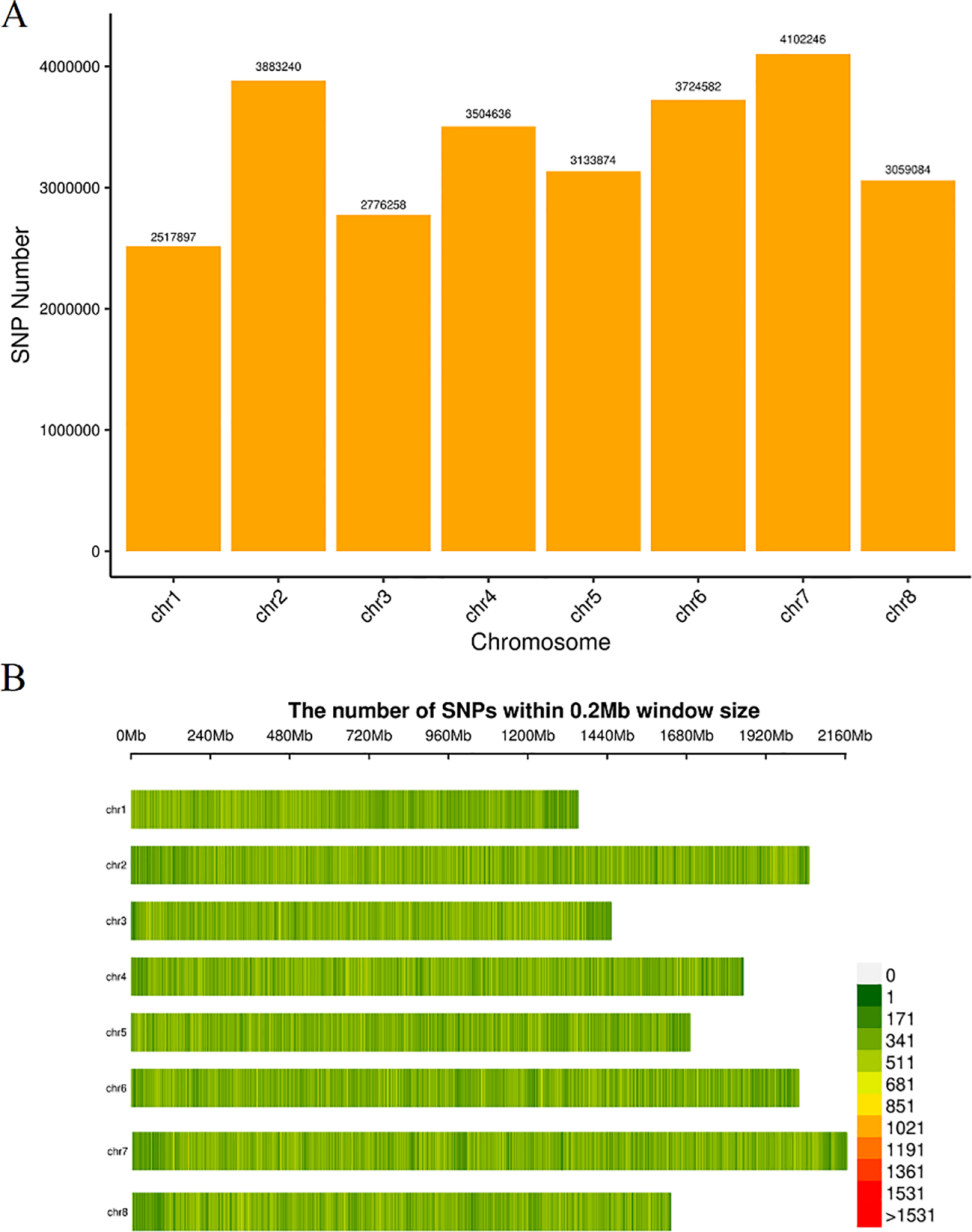
Single nucleotide polymorphism (SNP) identification of 77 garlic samples. **(A)** Number of SNPs on each chromosome. The horizontal axis represents the chromosome number, and the vertical axis represents the number of SNPs. **(B)** SNP density distribution on each chromosome. The horizontal axis represents the chromosome length, and the vertical axis represents the chromosome number. Different colors represent the number of SNPs in different regions. The color scale represents SNP counts within each 0.2 Mb window. Red indicates windows with SNP counts greater than 1531, while lighter colors represent lower SNP densities.

Using GATK software, a total of 26,701,817 raw SNP variants were identified across the 77 garlic accessions, distributed across all 8 chromosomes (**Figure 1A**). After flanking sequence conservation filtering, 4,759,583 SNPs with at least 50 bp conserved regions on both upstream and downstream sides were retained. Subsequent quality control filtering based on sequencing depth, genotype quality, missing rate, minor allele frequency, and biallelic status reduced the number to 100,743 high-confidence SNPs. To ensure locus specificity, 100 bp flanking sequences were aligned to the reference genome using BLAST, and only uniquely mapped loci were retained, resulting in 7,006 SNPs. A total of 4,297 SNP loci were identified as candidate loci with successful KASP primer design. Among these candidate loci, 30 generated reliable KASP genotyping data. From these successfully genotyped loci, 13 core SNP markers were finally identified for fingerprint analysis.

The SNP number and density on each chromosome were visualized in a chromosome-wise SNP distribution map (**Figure 1B**). The color gradient represents the number of SNPs per 0.2 Mb window, with warmer colors indicating higher SNP densities. Regions highlighted in red correspond to windows containing more than 1531 SNPs, whereas lighter green colors represent lower-density regions. Among them, chromosome chr7 harbored the highest number of SNPs, approximately 400,000, whereas chr1 exhibited the lowest SNP count, with around 250,000 SNPs. Chromosomes chr2, chr4, chr5, chr6, and chr8 displayed relatively similar SNP counts, each containing approximately 300,000 SNPs.

### 3.3 Core KASP-SNP marker selection and genetic diversity analysis

**Figure 2:**
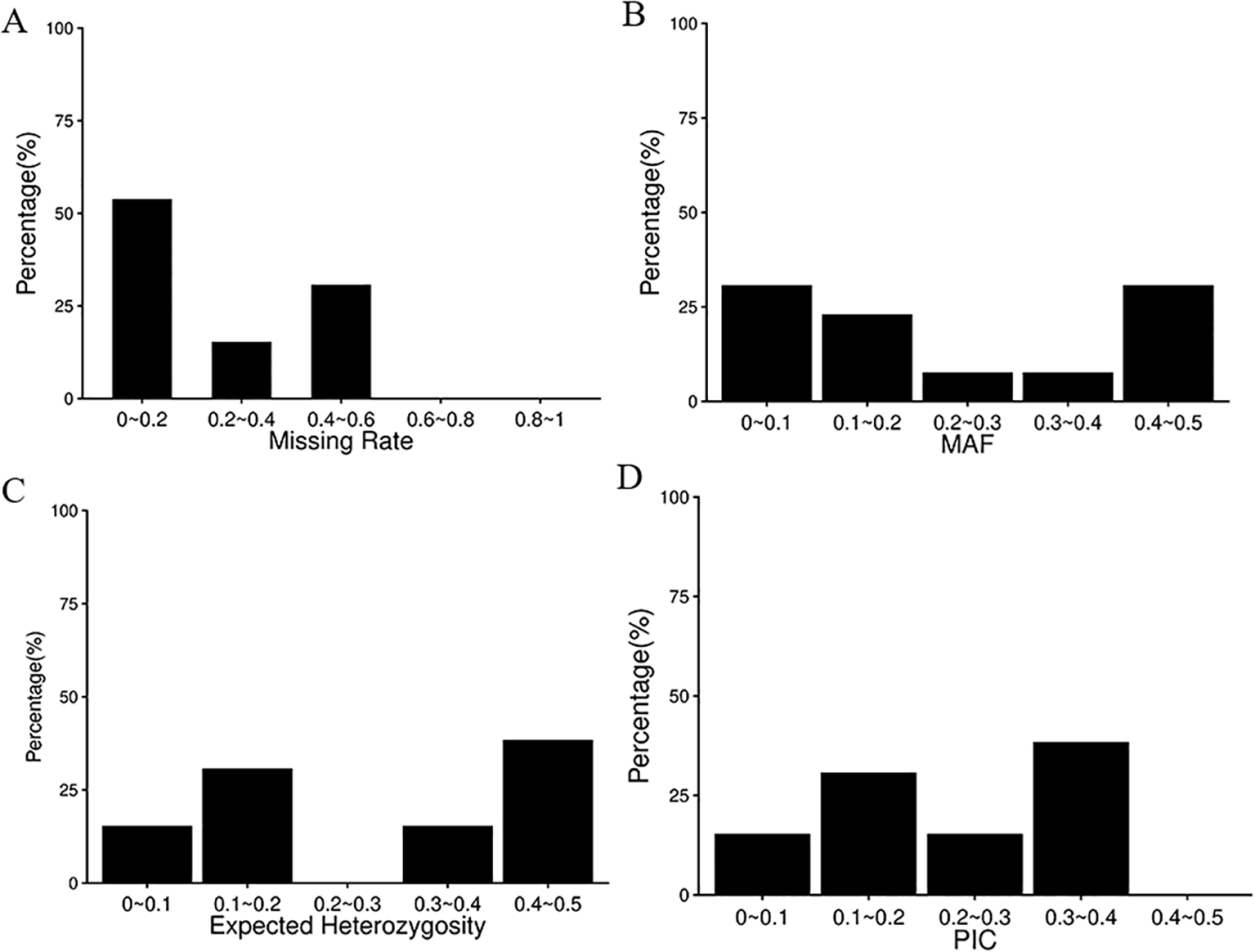
Percentage of missing rate, MAF, heterozygosity and PIC values for the 13 core KASP markers based on the data of 77 garlic varieties. PIC values were calculated based on KASP genotyping results across 77 garlic accessions. **(A)** Missing rate; **(B)** minor allele frequency (MAF); **(C)** expected heterozygosity; **(D)** polymorphism information content (PIC).

Based on the KASP validation results of 30 successfully genotyped markers, a total of 13 high-quality core SNP loci were identified as the minimal marker set for garlic variety discrimination. (**Table 1**). As shown in **Figure 2**, the missing rates of all 13 core KASP-SNP markers ranged from 0 to 0.6%. The average minor allele frequency (MAF) was 0.235, four markers (30.77%) had MAF values in the range of 0-0.1. The average expected heterozygosity (He) was approximately 0.281, with 5 markers (38.46%) showing He values between 0.4 and 0.5. The polymorphism information content (PIC) values were mainly distributed between 0.3 and 0.4, accounting for 38.46% of the markers, with an average PIC of approximately 0.227. To further evaluate the effectiveness of the selected markers, 13 core single nucleotide polymorphism (SNP) loci were applied for genotyping of 77 garlic varieties. The results showed that 73 accessions could be clearly distinguished, while the other four samples (136, 101, 11, and 103) shared identical multilocus genotypes with other samples under the final 13-locus combination, and thus could not be uniquely identified. These findings indicate that the selected 13-marker combination represents a minimal but efficient core SNP set obtained through stepwise screening of the validated KASP loci, and can be used for garlic variety identification.

**Table 1:** Statistics of 13 core SNP markers.

Number (N)	Number (N)	Observed number of alleles (Na)	Effective number of alleles (Ne)	Shannon’s information index (I)	Observed heterozygosity (Ho)	Expected heterozygosity (He)
chr3_252730951	75	2	1.942	0.678	0.453	0.485
chr5_969401364	75	2	1.987	0.69	0.093	0.497
chr8_1154271274	66	2	1.936	0.677	0.303	0.483
chr8_480253207	70	2	1.563	0.546	0.386	0.36
chr6_1165001766	77	2	1.862	0.655	0.078	0.463
chr5_73234679	76	2	1.17	0.276	0.132	0.145
chr5_831291085	75	2	1.204	0.31	0.027	0.169
chr1_983174590	76	2	1.216	0.322	0.092	0.178
chr1_885164743	77	2	1.053	0.12	0	0.051
chr2_372057825	75	2	1.436	0.481	0.373	0.304
chr1_1189998355	68	2	1.227	0.331	0	0.185
chr2_1134535486	76	2	1.027	0.07	0.026	0.026
chr1_439366133	75	2	1.999	0.693	0.973	0.5

N, number; Na, Observed number of alleles; Ne, Effective number of alleles; I, Shannon’s Information index; Ho, observed heterozygosity. Heterozygosity is the probability that the alleles in two randomly selected samples are different. He: Expected heterozygosity. The expected heterozygosity is the heterozygosity calculated based on theory.

### 3.4 Screening and validation of core SNP markers

Based on the GBS dataset, 4,297 SNP loci were identified as candidate loci with successful KASP primer design. These loci represented candidate markers for downstream validation rather than loci all having corresponding KASP genotyping data. Among them, 30 loci generated reliable and analyzable KASP genotyping results.

The 30 successfully genotyped loci were then subjected to stepwise evaluation based on genotyping quality and marker informativeness, including amplification stability, cluster clarity, missing rate, marker polymorphism, and discriminatory ability across the 77 garlic accessions. A subset of better-performing loci was first retained. Subsequently, during optimization of the minimal core marker combination for fingerprint construction, loci showing NN or missing genotype calls were excluded. As a result, 13 loci were finally selected as the core SNP marker set for downstream fingerprint construction and accession discrimination.

### 3.5 Population structure analysis and genetic diversity of garlic based on KASP-SNP markers

**Figure 3:**
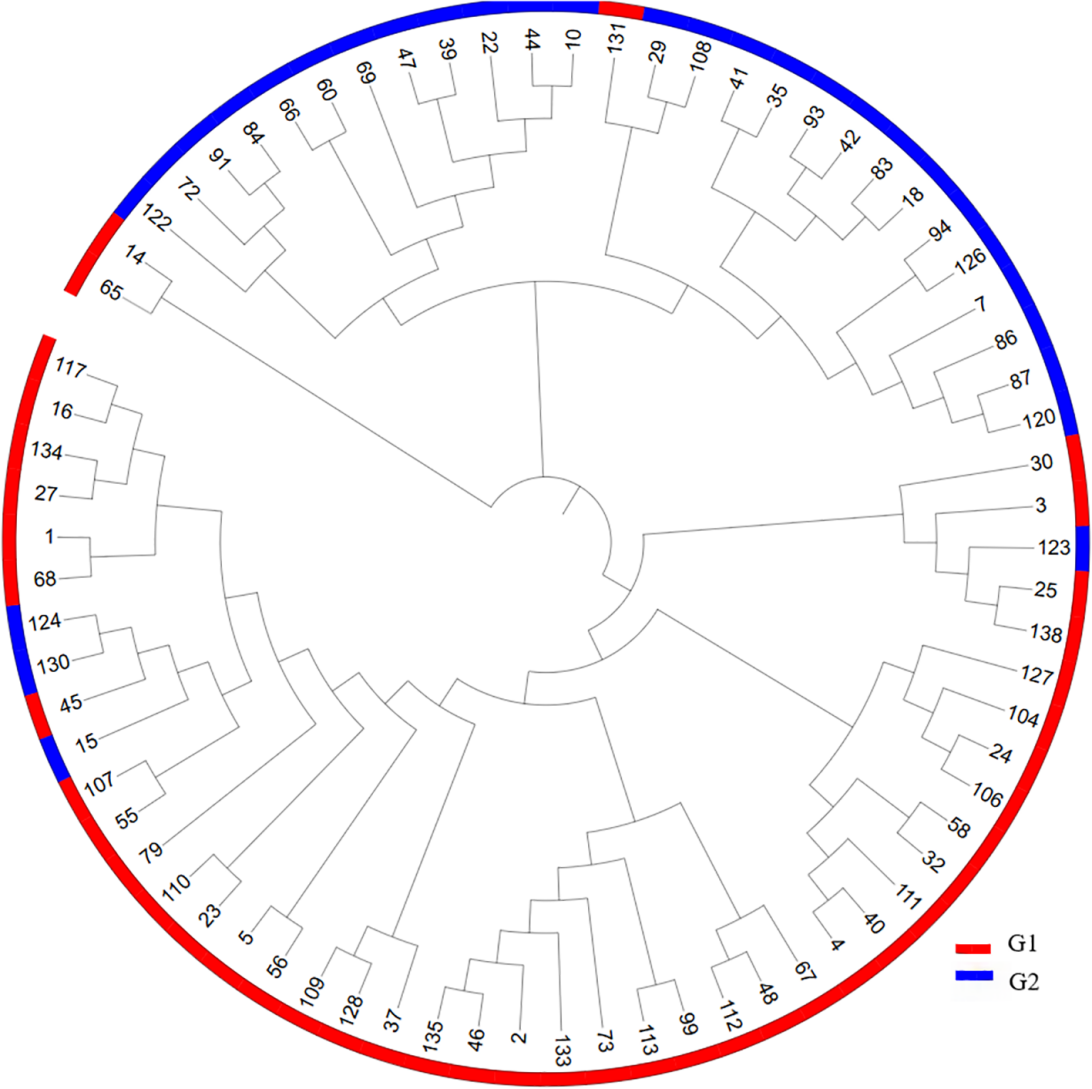
Cluster diagram of 73 garlic varieties.

A phylogenetic tree was constructed based on the genotyping data of 13 core SNP markers using the neighbor-joining method (**Figure 3**). As shown in **Figure 3**, Clustering analysis revealed that the 73 garlic accessions could be divided into two major groups, G1 (red) and G2 (green), each comprising accessions from multiple regions with complex origins. The G1 group, consisting of 42 accessions, included 9 from Jiangsu, 8 from Shandong, 4 from Henan, 3 from Yunnan, and 3 with unspecified provincial origins in China. Notably, all three garlic accessions from the Netherlands were grouped in G1. The G2 group contained 31 accessions, including 7 from Sichuan, 6 from Shandong, 4 from Yunnan, 1 from Gansu, 2 from foreign sources (Japan and Angola), and 6 from unspecified provinces in China, with others originating from Hunan, Hubei, and Xinjiang. The clustering results were consistent with the known genetic backgrounds of the accessions, with all Dutch garlic varieties clustering in G1 and most Sichuan accessions clustering in G2. Only one Sichuan accession was found in G1, suggesting that garlic varieties from similar or adjacent geographic regions tend to cluster together, thereby supporting the reliability of the sequencing data and the effectiveness of the SNP markers developed in this study.

**Figure 4:**
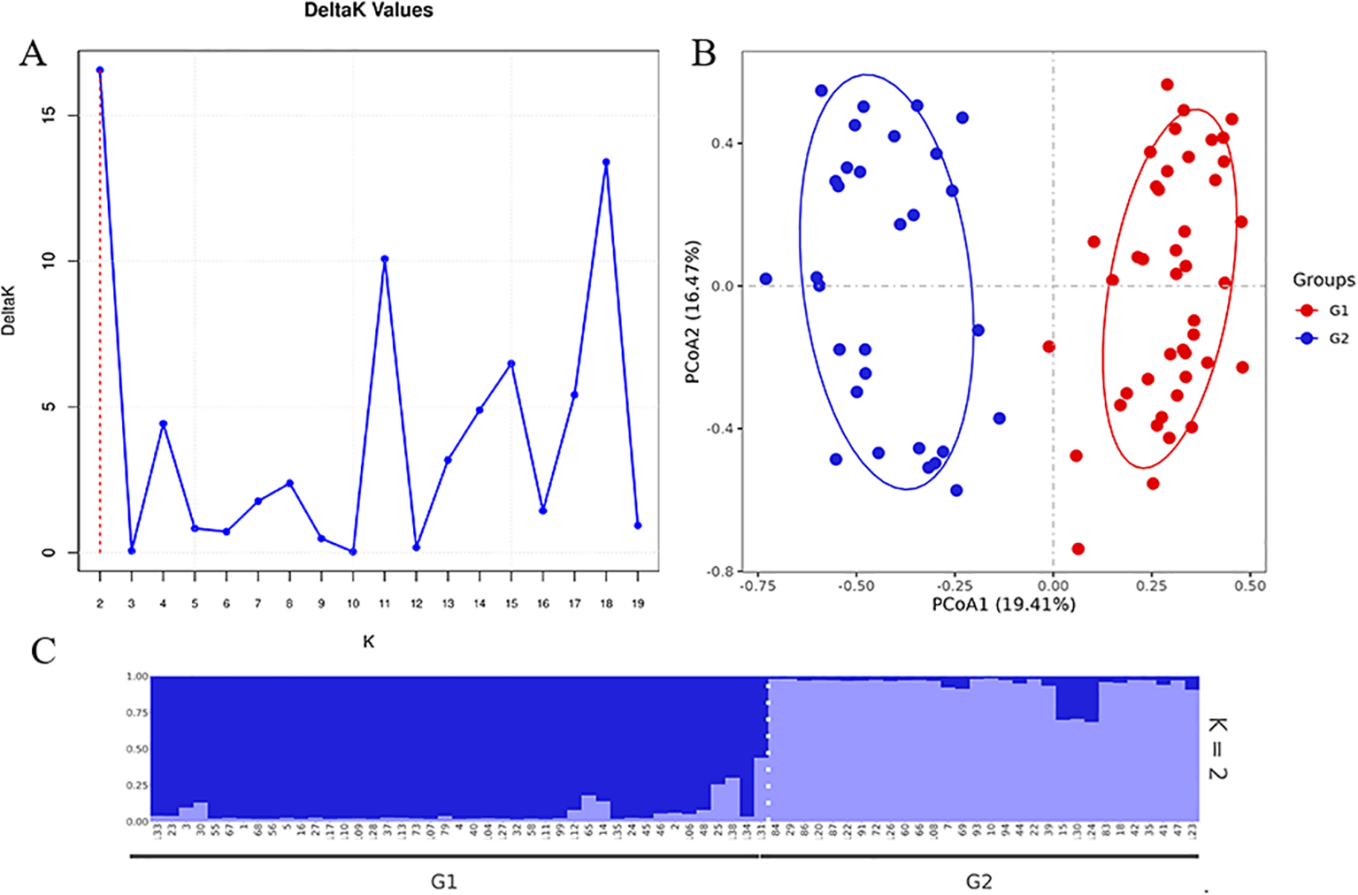
The judgment curve of optimal K value **(A)**, population structure **(B)** and PCA **(C)**diagram of 73 garlic varieties.

To further explore the population structure of garlic, STRUCTURE software was used to analyze genetic differentiation. The results showed that the highest ΔK value occurred when K = 2, indicating a relatively simple genetic structure among garlic varieties and limited gene flow, which is consistent with the clonal propagation characteristics of garlic (**Figure 4A**). The corresponding population structure assignment is shown in **Figure 4B**. Principal component analysis (PCA) further supported this division, with principal component 1 explaining 19.41% of the variation and principal component 2 explaining 16.47%. PC1 successfully separated the G1 and G2 groups, consistent with the clustering results (**Figure 4C**).

The 13 core SNP markers were primarily used for DNA fingerprint construction and accession discrimination. To obtain a more reliable assessment of population genetic structure and differentiation, additional analyses were conducted using 7,006 high-quality SNPs (**Supplementary Figure 1**). To obtain a more robust evaluation of population genetic structure, phylogenetic analysis, population structure analysis, and PCA were re-conducted using 7,006 high-quality SNPs derived from the GBS dataset. The phylogenetic tree divided the 73 garlic accessions into two major groups, designated G1 and G2 (**Supplementary Figure 1A**). PCA produced a consistent pattern, in which G1 and G2 were clearly separated, mainly along PC1, which explained 60.86% of the total variation, while PC2 explained 10.41% (**Supplementary Figure 1B**). These results indicate that the major genetic structure of the garlic panel is stable and is more reliably resolved using the 7,006-SNP dataset. Population structure analysis further supported this classification, with the optimal grouping observed at K = 2, where most accessions showed clear assignment to one of the two groups and only a few exhibited admixed ancestry (**Supplementary Figure 1C**).

Genetic differentiation analysis revealed a fixation index (Fst) of 0.099 between the G1 and G2 groups (**Table 2**), indicating a moderate level of genetic differentiation and suggesting that the two groups exhibited some degree of genetic divergence, although the differentiation was relatively limited. Meanwhile, the gene flow estimate (Nm) between the two groups was 2.275, which was greater than 1, indicating relatively frequent genetic exchange between them. A broader dataset containing 7006 high-quality SNP loci was used to recalculate the Fst value. The revised Fst value was 0.3556, and the Nm value was 0.45311(**Supplementary Table 4**).

**Table 2:** The details of Fst (above diagonal) and Nm (below diagonal) among subpopulations.

POP	G1	G2
G1	–	0.099
G2	2.275	–

### 3.6 Screening of optimal SNP markers and construction of DNA fingerprints for garlic germplasm resources

**Figure 5:**
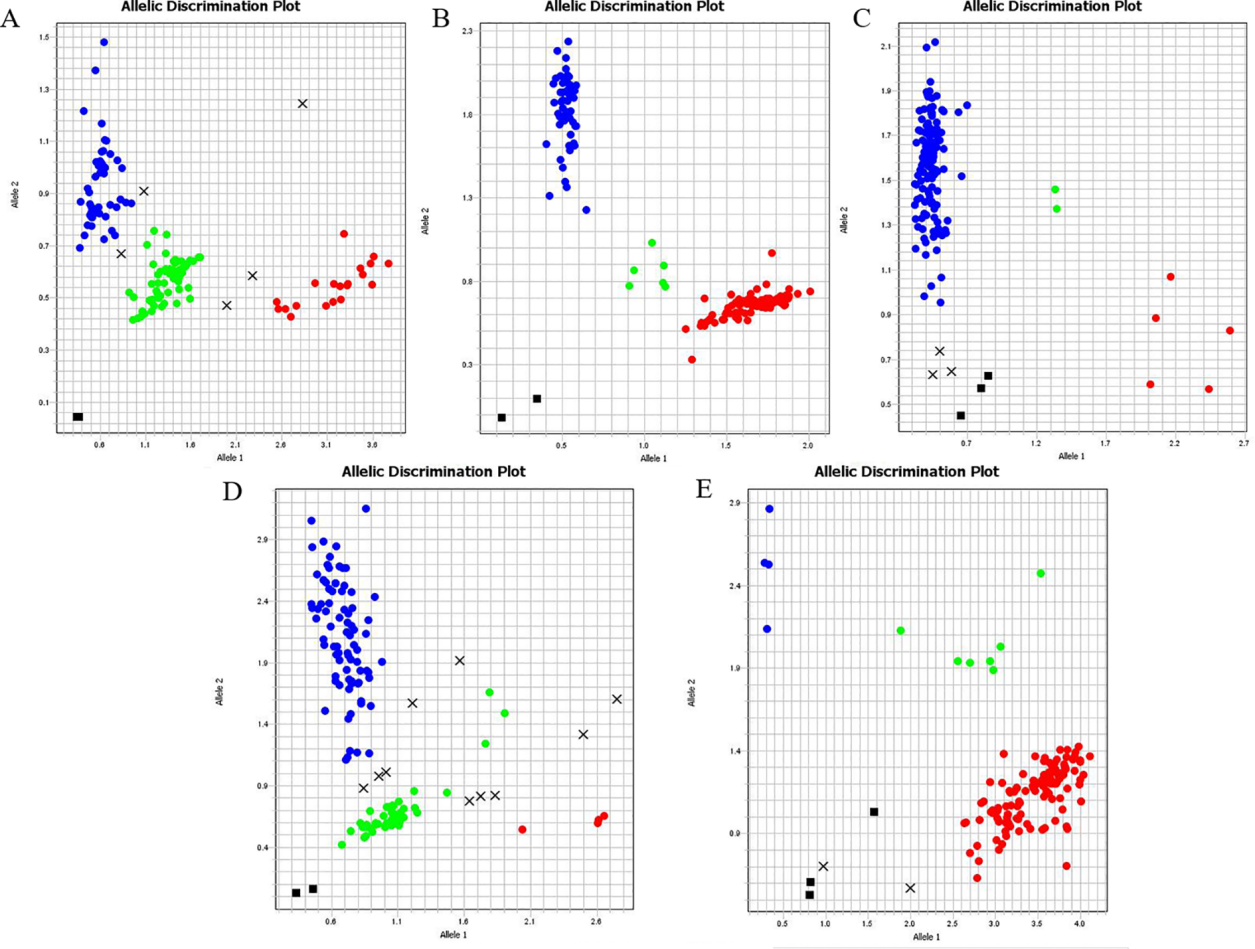
KASP verification results of some core SNPs. **(A)** Chr3-252730951. **(B)** Chr6-1165001766. **(C)** Chr5-831291085.**(D)** Chr8-480253207. **(E)** Chr1-983174590. Each dot in the figure represents a sample. Blue dots indicate samples homozygous for the HEX-labeled allele. Red dots indicate samples homozygous for the FAM-labeled allele. Green dots indicate heterozygous samples carrying both HEX and FAM labels. Black dots represent no-template controls (NTC).

**Figure 6:**
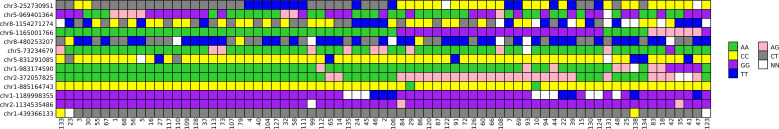
DNA fingerprint of the 73 distinguishable garlic accessions based on the 13 core KASP markers. Each dot in the figure represents a sample. Blue dots indicate samples homozygous for the HEX-labeled allele. Red dots indicate samples homozygous for the FAM-labeled allele. Green dots indicate heterozygous samples carrying both HEX and FAM labels. Black dots represent no-template controls (NTC).

From the 30 successfully genotyped KASP markers, a subset of loci with good genotyping quality and strong discriminatory power was first retained. After further optimization of the minimal marker combination for fingerprint construction, loci with NN/missing genotype calls in the final combination were excluded, and 13 loci were ultimately selected as the final core marker set. Using this final 13-locus set, multilocus genotypes were constructed for all 77 garlic accessions included in this study. The results showed that 73 accessions displayed unique multilocus genotypes, whereas 4 accessions could not be distinguished because they shared identical multilocus genotypes across all informative loci. These results indicate that the final 13-marker set provides effective discriminatory power for most garlic accessions, although it does not completely resolve all materials. Representative KASP genotyping results of several core loci are shown in **Figures 5A–E**. **Supplementary Figure 2** presents the unique multilocus genotype patterns identified among the distinguishable accessions, while **Figure 6**, **Supplementary Figure 3** summarize the fingerprint profiles of the 73 distinguishable accessions under the final 13-marker set.

**Figure 7:**
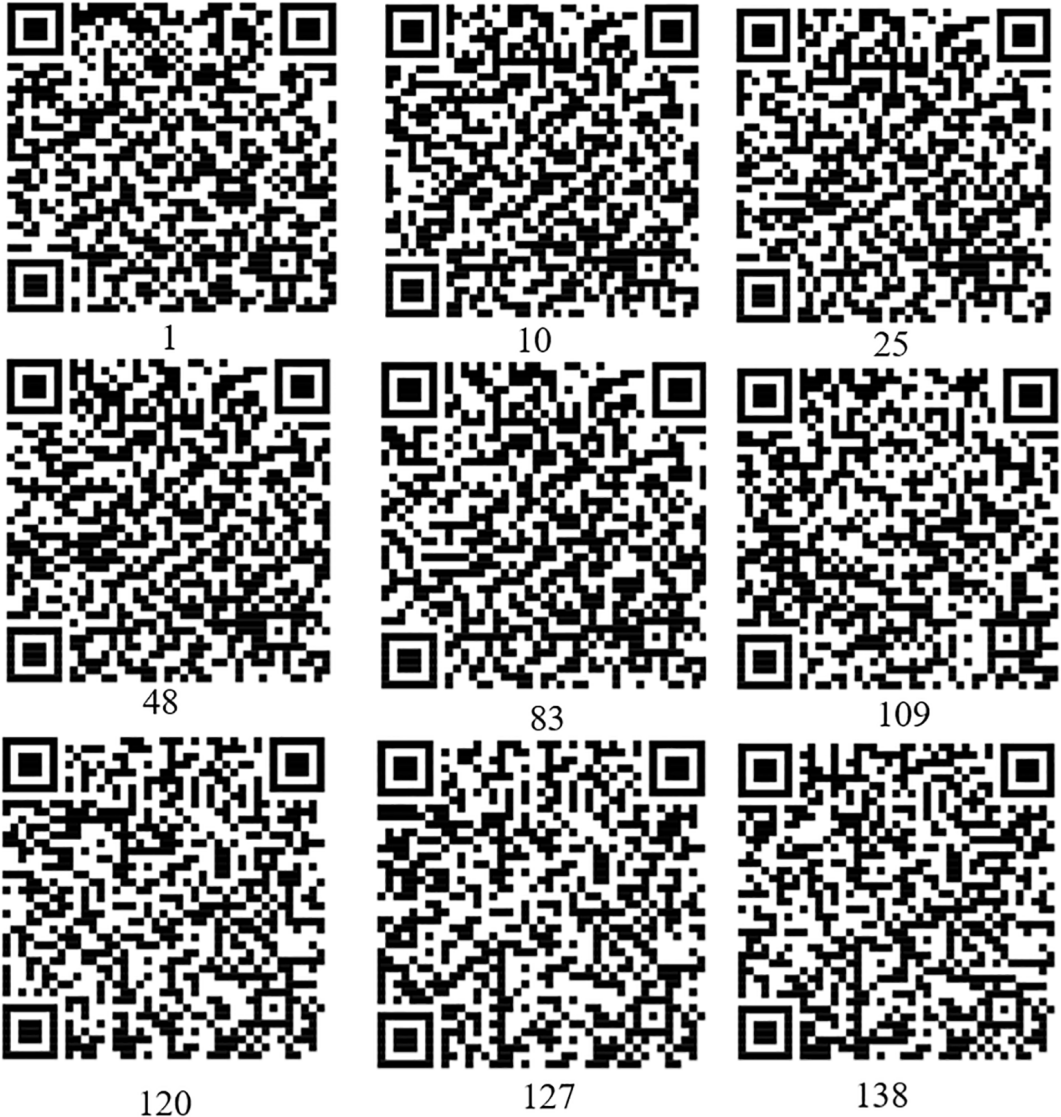
DNA fingerprint code of some varieties.

Among them, the two accessions with the greatest number of differing KASP markers were ‘Pin 307 non-bolting garlic’ (No. 47) and ‘Japanese flowering garlic’ (No. 123). To facilitate data accessibility and application, the fingerprint codes were further converted into QR codes. Representative DNA fingerprint codes for selected accessions are shown in **Figure 7**, while the complete fingerprint profiles of the 73 distinguishable accessions are provided in **Supplementary Figure 3**.

## 4 Discussion

### 4.1 Genetic diversity and population structure of garlic germplasm based on SNP markers

Due to long-term asexual reproduction and the influence of natural selection, the germplasm resources of garlic have undergone extensive variations (Ipek et al., 2003). The limited number of collected accessions and the lack of systematic studies on their genetic relationships have hindered germplasm innovation and breeding progress (Keller et al., 2013). Therefore, clarifying the genetic diversity and population structure of garlic germplasm is a prerequisite for effective resource utilization and breeding.

In this study, GBS technology was applied to 77 garlic accessions, generating a large number of genome-wide SNP loci. Population structure analysis based on core KASP-SNP markers revealed that the garlic accessions could be clearly divided into two major groups. This result was supported by phylogenetic tree construction, structure analysis and principal component analysis (PCA), indicating a relatively stable and reliable population structure. The optimal K value of 2 suggested a simple genetic structure, which is consistent with the clonal propagation characteristics of garlic and its limited gene flow among populations.

The Fst value between G1 and G2 was 0.099 and the Nm value was 2.275, indicating moderate genetic differentiation but substantial gene flow between the two groups. This pattern is consistent with the vegetative propagation of garlic, in which genetic variation mainly arises from somatic mutation and long-term artificial selection, while frequent germplasm exchange among regions maintains a shared genetic background. Therefore, although G1 and G2 could be separated into two genetic groups, they have not formed clear genetic isolation.

Differences in PIC values between GBS and KASP platforms are mainly attributable to their distinct genotyping principles (Ma et al., 2021). GBS is a sequencing-based method widely used for genome-wide SNP discovery, but genotype calling may be affected by uneven sequencing depth, missing data, and allele bias, particularly at low-coverage loci (Gardoce et al., 2023). In contrast, KASP is a locus-specific PCR-based genotyping method designed for targeted SNP validation, which generally provides more stable allele discrimination and lower missing rates (Wang et al., 2026). Therefore, discrepancies in genotype calls between the two platforms may lead to differences in allele frequency estimation and PIC values. In the present study, GBS was primarily used for SNP discovery, whereas KASP genotyping was employed to validate candidate loci and construct the garlic fingerprinting system.

### 4.2 Advantages of SNP markers and KASP technology for garlic variety identification

The commonly used methods for SNP detection and typing currently include whole-genome resequencing, simplified genome resequencing, and gene chip technology (Jehl et al., 2021). After the SNPs developed through these methods undergo variation detection, annotation, and screening, high-quality, representative, and highly discriminatory SNP markers with strong specificity and uniform distribution on the genome, as well as candidate markers for DNA fingerprinting patterns, are obtained (Deng et al., 2025). KASP can precisely perform biallelic typing of target SNPs on a wide range of genomic DNA samples, including complex genomic DNA samples (Zhao et al., 2025). Currently, has preserved over 600 garlic germplasm resources, but there are still deficiencies in the precise identification and utilization of garlic germplasm resources (Yang et al., 2024). Over the years, researchers have conducted various studies on the development of garlic molecular markers for germplasm resource identification. However, due to the lag in garlic genomics research, researchers mainly adopted traditional molecular markers.

KASP technology further enhances the applicability of SNP markers by offering a low-cost, high-throughput and gel-free genotyping platform (Rahman et al., 2023). It should be noted that the 4,297 loci reported in this study represent candidate loci with successful KASP primer design, rather than loci all having corresponding KASP genotyping data. Among these candidate loci, 30 generated reliable KASP genotyping data, and the final 13 core markers were identified from these successfully genotyped loci as the minimal marker set for fingerprint construction. The genome data of garlic is approximately 16Gb and it belongs to a species with a highly repetitive genome. Among the 7,006 candidate SNP loci in this study, KASP primers were successfully designed for 4,297 loci (61.3%). The primer design might fail due to the complex genome structure, multiple copy regions, high GC content, or the formation of secondary structures in the flanking sequences. Moreover, even the successfully designed primers might show lower amplification efficiency or unclear genotype clustering during the verification process. Therefore, the conversion rate observed in this study reflects the limitations of the technology and the genome rather than the limitations of the method. Compared with other SNP genotyping platforms, KASP technology is particularly suitable for large-scale variety identification and routine application due to its simplicity and efficiency (Qi et al., 2022). In this study, the large set of candidate SNPs was further reduced to a minimal panel of 13 core KASP markers, which could discriminate 73 out of 77 garlic accessions, demonstrating the high efficiency and practical applicability of KASP technology for garlic variety identification.

### 4.3 Construction, application value and limitations of SNP-based DNA fingerprinting in garlic

With the rapid development of sequencing technology and the significant reduction in sequencing costs, it has greatly facilitated the discovery of large-scale SNP loci in non-model species (Guo et al., 2021). Moreover, due to the wide distribution, stability, and high heritability of SNPs markers on the genome, they are currently widely used to construct DNA fingerprinting profiles (Roberts et al., 2018). DNA fingerprinting provides each germplasm accession with a unique molecular identity and plays an important role in variety identification, intellectual property protection and germplasm management (Hoque and Tasrin, 2018). In the present study, DNA fingerprint profiles were constructed based on 13 core KASP-SNP markers identified from 30 successfully genotyped KASP loci. These 13 markers represented a minimal core marker set with effective discriminatory power, rather than markers directly selected from all candidate loci at the primer-design stage. Although these 13 markers represented only a minimal core marker set selected from a large number of candidate loci, they were able to distinguish 73 of the 77 accessions. This result indicates that the developed marker set has high efficiency and practical value for garlic variety identification. Although there were four groups of samples that could not be distinguished, this indicates that these samples may have highly similar genetic backgrounds, or they may come from different sources but belong to the same variety. This is because in garlic, a plant that mainly reproduces asexually, the genetic variations among closely related samples are often limited due to its close genetic relationship.

The 13 core KASP-SNP markers were used to genotype 77 garlic accessions, of which 73 could be uniquely discriminated, while 4 remained indistinguishable. As the number of accessions increases, particularly when closely related or synonym varieties are included, additional SNP markers may be required to further improve identification resolution. Future studies should focus on expanding the garlic germplasm database and validating more KASP-SNP markers from the existing SNP resource pool. Overall, the SNP-based DNA fingerprinting system established in this study provides an efficient and reliable technical solution for garlic variety identification and lays a solid foundation for the protection and utilization of garlic germplasm resources.

## 5 Conclusion

This study demonstrates that the SNP markers developed through simplifying genomic sequencing combined with KASP genotyping technology provide an efficient and reliable approach for assessing the genetic diversity and population structure of garlic (*Allium sativum* L.). Using GBS-derived candidate SNP loci, 30 markers generated reliable KASP genotyping data, from which 13 core KASP-SNP loci were ultimately identified through stepwise screening of the 30 successfully genotyped KASP markers, based on genotyping quality, marker informativeness, and optimization of the minimal marker combination for fingerprint construction. Based on these 13 core markers, a SNP-based DNA fingerprinting system was established, which distinguished 73 of the 77 garlic accessions included in this study. The KASP-SNP markers developed in this study provide practical molecular tools for garlic variety identification, germplasm evaluation, and intellectual property protection, and offer a reference for SNP gene fingerprint analysis of other vegetatively propagated crops.

## Data Availability

The datasets presented in this study can be found in online repositories. The names of the repository/repositories and accession number(s) can be found below: https://www.ncbi.nlm.nih.gov/, PRJNA1313812.
