# Supplementary material for: Development of KASP molecular markers and fingerprinting based on reduced representation genome sequencing of garlic
Source: Front Plant Sci. 2026 May 8;17:1785664. doi: 10.3389/fpls.2026.1785664 (PMC13194573; doi:10.3389/fpls.2026.1785664)
Supplement: Supplementary file 1 [file DataSheet1.pdf]

Supporting Information for

Development of KASP molecular markers and fingerprinting based on reduced representation genome sequencing of garlic

Qingqing Yang[1, +], Jide Fan[1, +], Xiaohui Song,Yongqiang Zhao, Biwei Zhang, Xinjuan Lu, Jie Ge, Canyu Liu, Mengqian Li, Guangyang Liu, Yan Yang, Yi Feng, Feng Yang*

Xuzhou Institute of Agricultural Sciences in Jiangsu Xuhuai District, Key Laboratory of Biology and Genetic Breeding of Sweetpotato, Ministry of Agriculture and Rural Affairs National Agricultural Experimental Station for Soil Quality, Xuzhou 221121, China

\+ These authors contributed equally to this work.

\

Table S1 Garlic germplasm materials and sources

ID	Variety name	Country	Province	Classification	ID	Variety name	Country	Province	Classification
1	Shunhebendisuan	China	Jiangsu	White-skinned	69	Sheyang White-skinned Garlic	China	Jiangsu	White-skinned
2	Xizangdutouhongsuan	China	Tibet	White-skinned	72	Chaling Purple-skinned Garlic	China	Hunan	Purple-skinned
3	Nianzhuangzipisuan	China	Shandong	Purple-skinned	73	Harbin Acheng Garlic	China	Heilongjiang	Purple-skinned
4	Lanzhou Garlic	China	Gansu	White-skinned	79	Zigong White Garlic	China	Sichuan	White-skinned
5	Xiangfu Red-skinned Garlic	China	Henan	Red-skinned	83	Laiwu Red-skinned Garlic B	China	Shandong	Red-skinned
7	Chengdu Single-clove Garlic	China	Sichuan	White-skinned	84	Linyi No.2 Garlic	China	Shandong	White-skinned
10	Danie	China	Details unknown	White-skinned	86	Du (Single-clove Garlic)	China	Details unknown	White-skinned
11	Jinxiang Red-Garlic A	China	Shandong	Red-skinned	87	Zigong Bolt Garlic B	China	Sichuan	White-skinned

14	Zhongmu White Garlic	China	Henan	White-skinned	91	Yunnan Shanyao Garlic	China	Yunnan	Purple-skinned
15	Gansu-1	China	Gansu		93	Ershui Early-maturing Garlic-2	China	Sichuan	Purple-skinned
16	Weifang White Garlic	China	Shandong	White-skinned	94	Yuanmou Small Garlic	China	Yunnan	Purple-skinned
18	Line 258 Hard-leaf Garlic	China	Sichuan	Purple-skinned	99	Jiaxiang Bolting-free Garlic	China	Shandong	White-skinned
22	Cangshan Dapuye Garlic	China	Shandong	White-skinned	101	Zhongmu Garlic	China	Henan	White-skinned
23	Yuanmou Garlic-3	China	Yunnan	White-skinned	103	Bolt Garlic-1	China	Shandong	Purple-skinned
24	Jinhong No.6	China	Shandong	Red-skinned	104	Yunnan Tou Garlic No.8	China	Yunnan	White-skinned
25	Jinfeng No.2	China	Shandong	White-skinned	106	Space Garlic No.1	China	Jiangsu	Purple-skinned
27	Tibetan Single-White Garlic	China	Tibet	White-skinned	107	Qinghong Early Garlic	China	Jiangsu	Red-skinned

29	Linyi No.1 Garlic	China	Shandong	White-skinned	108	Guanghan White Garlic	China	Sichuan	Red-skinned
30	Netherlands-4	Netherlands	-	White-skinned	109	Heze Garlic	China	Shandong	Purple-skinned
32	Hunyuan Garlic-1	China	Shaanxi	Purple-skinned	110	Pingyi Garlic	China	Shandong	White-skinned
35	Line 259 Hard-leaf Garlic	China	Details unknown	White-skinned	111	Shangnan Black-skinned Garlic	China	Henan	Black-skinned
37	Netherlands-6	Netherlands	-	White-skinned	112	Yulin Large White Garlic B	China	Guangxi	White-skinned
39	Bolt Garlic-5	China	Details unknown	White-skinned	113	Tibetan Vegetable Garlic	China	Tibet	White-skinned
40	Yongsheng Sina Garlic	China	Details unknown	White-skinned	117	Enshi Red Garlic	China	Hubei	Purple-skinned
41	Yuanmou Single Purple Garlic	China	Yunnan	Purple-skinned	120	Xiangfu Purple-skinned Garlic	China	Henan	Purple-skinned
42	Yuanmou Purple Garlic-2	China	Yunnan	Purple-skinned	122	Ningque Red Garlic	China	Shandong	Red-skinned
44	Yelu Garlic	China	Shandong	White-skinned	123	Japanese Flowering Garlic	Japan	-	White-skinned

45	Hanfeng Early Garlic	China	Jiangsu	White-skinned	124	Xinjiang Purple-skinned Garlic (2018)	China	Xinjiang	Purple-skinned
46	Tianshan Garlic-1	China	Xinjiang	White-skinned	126	Baotou Haidai Garlic	China	Inner Mongolia	Purple-skinned
47	Line 307 Non-bolting Garlic	Angola	-	Purple-skinned	127	Jimusar Garlic	China	Xinjiang	Purple-skinned
48	Netherlands-5	Netherlands	-	White-skinned	128	Weifang Garlic	China	Shandong	White-skinned
55	Enshi White Garlic	China	Hubei	White-skinned	130	Minle Purple Garlic	China	Sichuan	Purple-skinned
56	Dongdongqingbai Garlic	China	Henan	White-skinned	131	Garlic (Netherlands origin)-2	Netherlands	-	Purple-skinned
58	Pizhou Purple Garlic	China	Jiangsu	Purple-skinned	133	Xusuan 917	China	Jiangsu	Purple-skinned
60	Zigong Bolt Garlic A	China	Sichuan	White-skinned	134	Xusuan 918	China	Jiangsu	Purple-skinned
65	Laiwu Red-skinned Garlic B	China	Shandong	White-skinned	135	Xusuan 815	China	Jiangsu	White-skinned

66	Cuiyu Garlic	China	Details unknown	White-skinned	136	Xubai No.1	China	Jiangsu	White-skinned
67	Yulin Large White Garlic C	China	Guangxi	White-skinned	138	E8 Irradiated Garlic	China	Jiangsu	Purple-skinned
68	Yuanmou Garlic	China	Yunnan	White-skinned					

Table S2 Primers used for the initial screening validation of candidate SNP loci.

Number	SNP Position	Forward primer (5′-3′)	Reverse primer (5′-3′)
1	chr3_252730951	ccacagcagcatttattctggcat	attggagttggactttctcccaat
2	chr5_969401364	cttgtacattaccgttctgtttccat	gatgcggagactgagaaggaatgatt
3	chr8_1154271274	caagccctagtccttgtgaccaa	gtcggaagtggagtaacttggat
4	chr8_480253207	cacgccctatgagcatactaagaaatttg	ccttttctttccttatttcaactgagacc
5	chr6_1165001766	gcctgcgaaaatcaaagaaagc	gacgaagaacgcaaggcagttg
6	chr5_73234679	atattctaaacaagctgtgagggcat	ctgtctcagtataacacacctcccac
7	chr5_831291085	ctccctttcctacttatgttgtccata	gaactaccctacccatgattcagagtt
8	chr1_983174590	tctgctatctagtatttgagttaccact	cagccttggataacaccataagaattta
9	chr1_885164743	acgatctttatgggagttaccagag	acaagctcttcccttattgcaaggt
10	chr2_372057825	tgaatccatcgttaaccgtatgact	ctcgagaaggtgcttgagcttatga
11	chr1_1189998355	catctctaagacttagtggttttctagc	gaaacaaaccaatgttcgatagtccgatt

12	chr2_1134535486	tgatcttttgatggttttgggtgg	agaaaataagggggaaggcctcac
13	chr1_439366133	ggataaaggtagttaggtagatgaactt	gttcccagttgtagatttatgagtcattc。

Table S3 Quality assessment of sequencing data for 77 varieties of garlic

sample	Clean_Data	GC(%)	Depth	Mapping rate(%)
101	8411491259	37.64	5.74924	99.25
103	8854091990	37.37	6.05175	98.71
104	8195343134	37.18	5.6015	99.33
106	15373626313	37.58	10.5078	99.28
107	9804094174	37.1	6.70108	99.34
108	5732668778	37.12	3.91827	98.7
109	9952670630	37.82	6.80263	99.29
10	13090000457	37.8	8.94699	98.66
110	8219276070	37.89	5.61786	99.27
111	23500181464	37.5	16.0623	99.29
112	5947777439	37.52	4.06529	99.3
113	12148597576	37.52	8.30354	99.3
117	12977508227	37.83	8.8701	99.25
11	9896551115	37.67	6.76427	99.32
120	16654034610	37.79	11.383	98.68
122	7877671369	36.64	5.38437	98.7
123	10327811807	37.27	7.05904	83.77

124	8582601245	37.57	5.86619	98.59
126	7837023809	37.16	5.35659	98.7
127	6494825057	37.28	4.4392	99.16
128	8119772932	37.16	5.54985	99.23
130	9990629831	37.78	6.82857	98.69
131	5996302991	37.76	4.09846	98.92
133	23269145966	37.74	15.9044	99.29
134	16843564649	37.77	11.5125	99.32
135	12631579752	37.75	8.63366	99.28
136	6943517672	37.9	4.74588	99.26
138	7761988299	37.4	5.3053	90.91
14	9572768757	38.17	6.54297	99.31
15	16053746270	38.62	10.9727	98.75
16	7041631730	38.39	4.81294	99.31
18	18146873114	38.06	12.4033	98.5
1	21080172178	37.97	14.4083	99.29
22	11907245766	38.03	8.13858	98.7
23	12945579809	38.15	8.84827	99.37
24	19335634038	37.87	13.2159	99.34
25	7050483462	38.12	4.81899	99.03
27	7938930205	37.81	5.42624	99.01
29	9788905114	37.92	6.69069	98.38
2	17360460815	38.03	11.8658	99.31
30	8196020993	38.04	5.60196	99.13

32	9110186865	38.4	6.22679	98.98
35	7479052434	38.31	5.11192	98.174
37	13317777966	38.65	9.10267	99.17
39	19654850868	38.14	13.434	98.44
3	10720074110	37.63	7.32715	99.23
40	9721844239	38.18	6.64486	99.15
41	21507879036	38.11	14.7006	98.43
42	9384205805	38.17	6.41408	98.47
44	20502438102	38.3	14.0134	98.59
45	17005220493	38.04	11.623	99.23
46	15491263367	38.1	10.5882	99.2
47	18493253764	37.66	12.6401	98.4
48	9081921854	37.4	6.20747	99.26
4	17135425727	37.66	11.712	99.29
55	21571926726	37.94	14.7444	99.34
56	8746933895	38.01	5.97851	99.28
58	8535104009	36.52	5.83372	99.32
5	17455247237	37.93	11.9306	99.27
60	14731407083	37.52	10.0689	98.76
65	15951935881	38.72	10.9031	99.19
66	9824669546	38.3	6.71514	98.64
67	6143572960	38.03	4.19912	99.22
68	10352481984	37.92	7.0759	99.2
69	10263518750	37.77	7.01509	98.53

72	14410389122	38.06	9.84947	98.46
73	11394948234	35.98	7.78842	99.13
79	8185875294	37.03	5.59503	99.79
7	14220288597	37.9	9.71954	98.65
83	10732835637	36.62	7.33587	98.65
84	5591099161	36.65	3.8215	98.67
86	9861031116	37.39	6.73999	98.65
87	5707173109	37.39	3.90084	98.6
91	8046996990	37.74	5.5001	98.58
93	15022314776	36.09	10.2677	98.36
94	8744601193	37.19	5.97692	98.52
99	16046573238	36.67	10.9678	99.09

Table S4 Fst (above diagonal) and Nm (below diagonal) among subpopulations based on 7006 SNPs

POP	G1	G2
G1	-	0.3556
G2	0.45311	-

Table S5 Genotype matrix of 73 garlic accessions based on 13 KASP markers

Sample	Chr1-1189998355	Chr2-372057825	Chr1-439366133	Chr1-885164743	Chr1-983174590	Chr2-1134535486	Chr3-252730951	Chr5-73234679	Chr5-969401364	Chr6-1165001766	Chr8-480253207	Chr8-11542712[illegible]
104	GG	AA	TC	CC	AA	GG	TT	AA	AA	AA	TT	CC
106	TT	AA	TC	CC	AA	GG	TT	AA	AA	AA	NN	CC
107	GG	AA	TC	CC	AA	GG	TC	AA	AA	AA	CT	CT
108	GG	AG	TC	CC	AA	GG	CC	AG	GA	GG	NN	NN
109	GG	AA	TC	CC	AA	GG	TC	AA	GG	AA	TT	CC
10	NN	AG	TC	AA	AA	GG	CC	AA	AA	GG	TT	NN
110	GG	AA	TC	CC	AA	GG	TC	AA	GG	AA	NN	CC
111	GG	AA	TC	CC	AA	GG	TT	AA	GG	AA	CT	CT
112	GG	AA	TC	CC	AG	GG	TC	AA	AA	AA	TT	CC
113	GG	AA	TC	CC	AA	GG	TC	AG	AA	AA	TT	CT
117	GG	AA	TC	CC	AA	GG	TC	AA	GG	AA	CT	CT
120	GG	AG	TC	CC	AA	GG	CC	AA	AA	GG	CT	CC

122	GG	AG	TC	CC	AA	GG	NN	AA	AA	GG	TT	CC
123	GG	AA	NN	CC	AA	GG	CC	AA	GG	GG	TT	NN
124	NN	AA	TC	CC	AA	GG	TT	AA	GG	GG	TT	CT
126	GG	AG	TC	CC	AA	GG	CC	AA	GG	GG	CT	CT
127	GG	AA	TC	CC	AA	GG	TT	AA	AA	AA	TT	CT
128	GG	AA	TC	CC	AA	GG	TC	AA	GG	AA	TT	CT
130	NN	AA	TC	CC	AA	GG	TC	AA	GG	GG	CT	TT
131	GG	AG	TC	CC	AA	GG	CC	AG	NN	AA	CT	NN
133	GG	AA	CC	CC	AA	GG	TC	AG	GG	AA	CT	NN
134	GG	AA	TC	CC	AA	GG	TC	AA	AA	AA	CT	CC
135	NN	AA	TC	CC	AA	GG	TC	AG	GG	AA	CT	CT
138	TT	AA	CC	CC	NN	GG	CC	AA	GG	AA	TT	NN
14	GG	AG	TC	CC	AA	GG	TC	AA	GG	AA	TT	CT
15	GG	AA	TC	CC	AA	GG	TC	AA	AA	GG	CT	CC

16	GG	AA	TC	CC	AA	GG	TC	AA	GG	AA	CT	CC
18	GG	AG	TC	CC	AG	GG	CC	AA	GG	AG	TT	NN
1	GG	AA	TC	CC	AA	GG	TC	AA	GA	AA	CT	CC
22	TT	AG	TC	CC	AA	GG	CC	AA	AA	GG	TT	TT
23	GG	AA	NN	CC	AA	GG	TC	AA	GG	AA	CC	TT
24	NN	AA	TC	CC	AA	GG	TT	AA	AA	AA	TT	TT
25	GG	AA	TC	CC	AG	GG	CC	AA	GG	AA	TT	NN
27	GG	AA	TC	CC	AA	GG	TC	AA	GG	AA	CT	CC
29	GG	AG	TC	AA	AA	GG	CC	AG	GG	GG	TT	CC
2	TT	AA	TC	CC	AA	GG	TC	AG	GG	AA	TT	TT
30	GG	AA	TC	CC	AA	GG	CC	AA	AA	AA	TT	TT
32	GG	AA	TC	CC	AA	GG	TT	AA	GA	AA	CT	CC
35	GG	NN	TC	CC	GG	AG	CC	AA	AA	AG	CT	TT
37	GG	AA	TC	CC	AA	GG	TC	AA	GG	AA	TT	TT

39	TT	AG	TC	CC	AA	GG	TT	AA	GG	GG	TT	NN
3	GG	AA	TC	CC	AA	GG	CC	AA	GG	AA	TT	CT
40	GG	AA	TC	CC	AA	GG	TT	AA	AA	AA	CT	CT
41	GG	NN	TC	CC	GG	GG	CC	AA	GG	AG	CT	TT
42	GG	AG	TC	CC	GG	GG	CC	GG	AA	AG	NN	CC
44	NN	AG	TC	CC	AG	GG	TT	AA	AA	GG	TT	CT
45	NN	AA	TC	CC	AA	GG	TC	AA	AA	AA	CT	TT
46	TT	AA	TC	CC	AA	GG	TC	AG	AA	AA	CT	TT
47	TT	AG	TC	CC	GG	GG	NN	AA	GG	AG	TT	TT
48	NN	AA	TC	CC	AG	GG	TC	AA	AA	AA	TT	TT
4	GG	AA	TC	CC	AA	GG	TT	AA	AA	AA	CT	CC
55	GG	AA	TC	CC	AA	GG	TC	AA	AA	AA	CT	CC
56	GG	AA	TC	CC	AA	GG	TC	AA	GA	AA	TT	CC
58	GG	AA	TC	CC	AA	GG	TT	AA	GA	AA	TT	CT

5	GG	AA	TC	CC	AA	GG	TC	AA	GA	AA	TT	TT
60	GG	AG	TC	CC	AA	GG	CC	AA	GG	GG	TT	CC
65	GG	AG	TC	CC	AA	GG	TC	AA	GG	AA	TT	CC
66	GG	AG	TC	CC	AA	GG	CC	AA	GG	GG	TT	CT
67	GG	AA	TC	CC	AA	GG	TC	AA	AA	AA	TT	CT
68	GG	AA	TC	CC	AA	GG	TC	AA	GA	AA	CT	CT
69	GG	AG	TC	CC	AA	GG	TT	AA	GG	GG	TT	CC
72	GG	AG	TC	CC	AA	GG	CC	AA	AA	GG	TT	CT
73	GG	AA	TC	CC	AA	GG	TC	AG	GG	AA	TT	CC
79	GG	AA	TC	CC	AA	GG	TC	AA	AA	AA	CC	CC
7	GG	AG	TC	CC	AA	GG	TT	AA	AA	GG	CT	TT
83	GG	AG	TC	CC	AG	GG	CC	AA	AA	AG	TT	CC
84	GG	AG	TC	CC	AA	AG	CC	AA	AA	GG	TT	CT
86	GG	AG	TC	CC	AA	GG	CC	AA	AA	GG	CC	CC

87	GG	AG	TC	CC	AA	GG	CC	AA	AA	GG	CT	CT
91	GG	AG	TC	CC	AA	GG	CC	AA	AA	GG	TT	CC
93	GG	AG	TC	CC	AG	GG	CC	NN	AA	GG	NN	CC
94	NN	AG	TC	CC	AA	GG	CC	AA	GG	GG	CT	TT
99	GG	AA	TC	CC	AA	NN	TC	AG	AA	AA	NN	CC

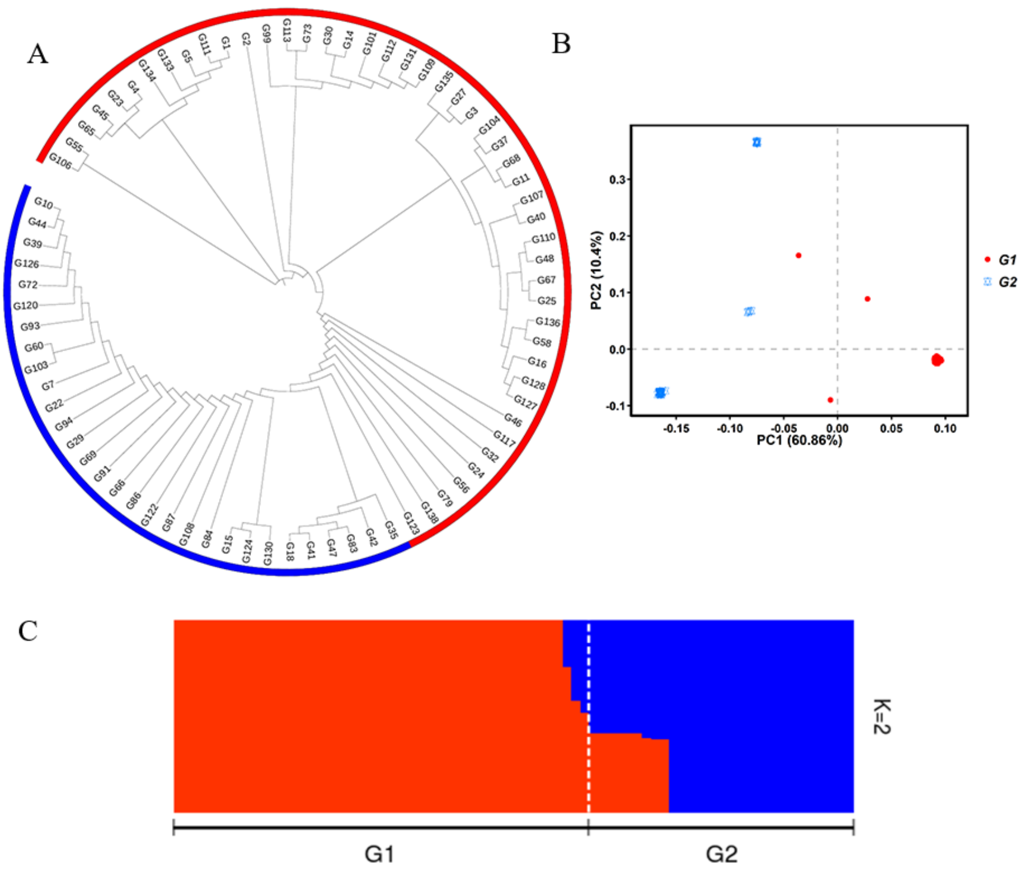

Figure S1. Re-evaluation of population genetic structure of 77 garlic accessions based on 7,006 high-quality SNPs (A) Phylogenetic tree showing the division of accessions into two major groups, G1 and G2. (B) Principal component analysis (PCA) of the 77 garlic accessions. (C) Population structure analysis at K = 2.

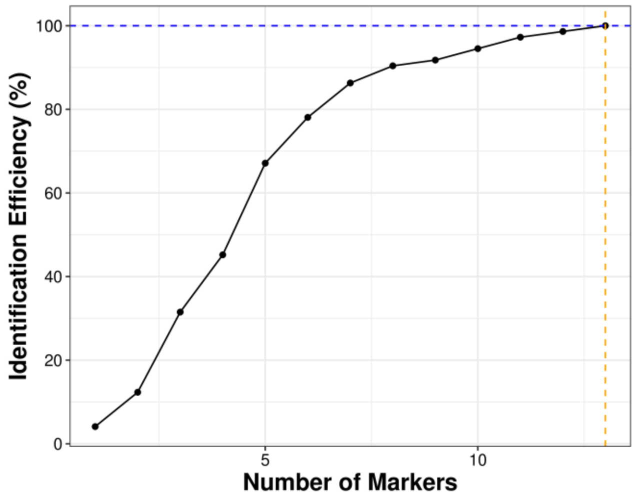

Figure S2 Multilocus genotype patterns identified using the final 13 core KASP markers. A total of 73 unique multilocus genotypes were obtained among the 77 garlic accessions included in this study, whereas 4 accessions remained unresolved because they shared identical multilocus genotypes with other accessions under the final marker set. These unresolved accessions were retained in the analysis and were not excluded from the dataset.

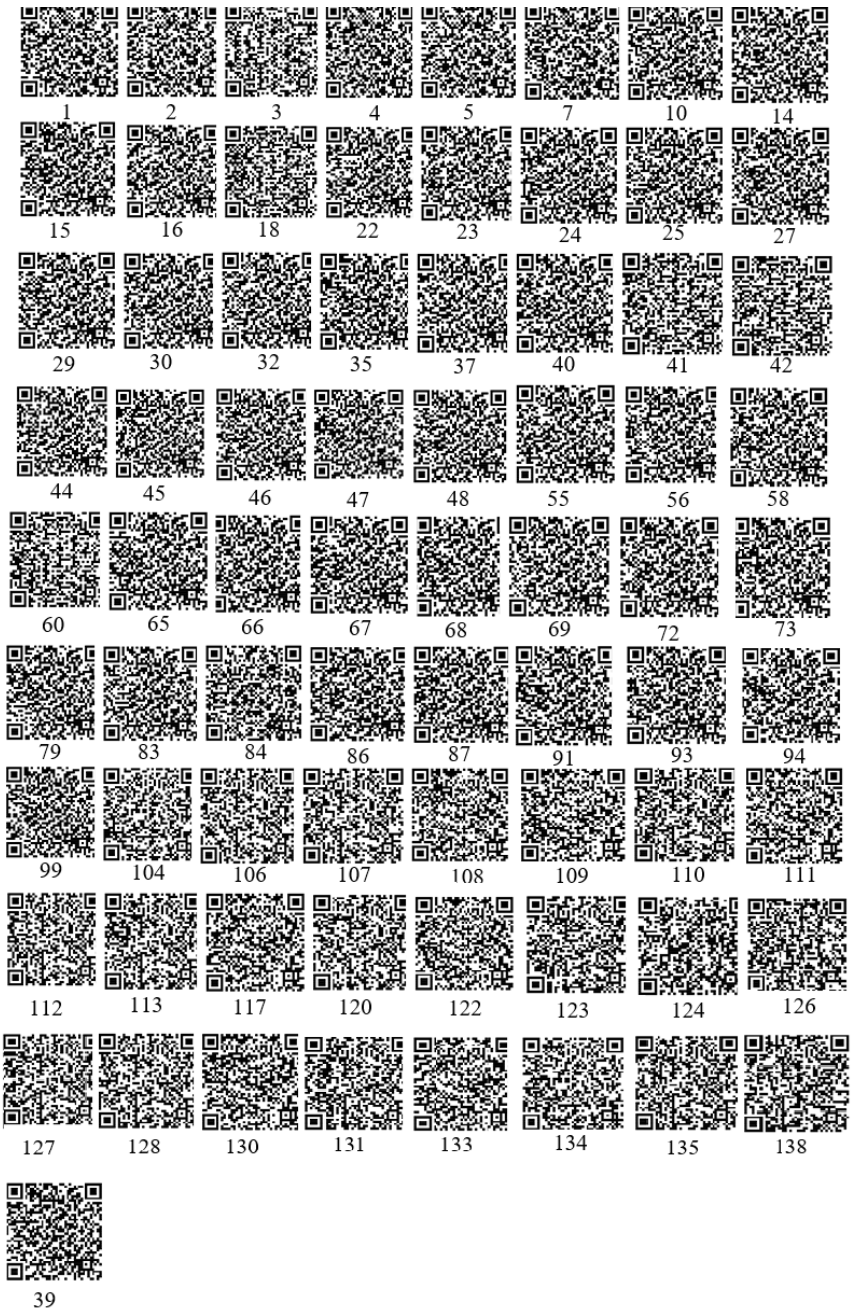

Figure S3 Complete fingerprint profiles of the 73 distinguishable garlic accessions based on the final 13 core KASP markers. These accessions represent the subset of the 77 analyzed accessions that displayed unique multilocus genotypes under the final marker set.
